# Randomized clinical trial of tissue equivalent bolus prescription in postmastectomy radiotherapy stratified by skin involvement status

**DOI:** 10.1016/j.ctro.2022.100570

**Published:** 2022-12-21

**Authors:** Lucas Gomes Sapienza, Maria Aparecida Conte Maia, Maria José Leite Gomes, André Mattar, Glauco Baiocchi, Vinicius Fernando Calsavara

**Affiliations:** a Department of Radiation Oncology, Dan L Duncan Comprehensive Cancer Center, Baylor College of Medicine, Houston, TX, USA; b Department of Radiation Oncology, São Camilo Oncologia, São Paulo, SP, Brazil; c Department of Radiation Oncology, Hospital Federal dos Servidores do Estado (HFSE-RJ), Rio de Janeiro, RJ, Brazil; d Department of Mastology, Hospital Pérola Byington, São Paulo, SP, Brazil; e Department of Gynecologic Oncology, A.C. Camargo Cancer Center, São Paulo, SP, Brazil; f Department of Biostatistics and Bioinformatics, Samuel Oschin Cancer Center, Cedars-Sinai, Los Angeles, CA, USA

**Keywords:** Breast cancer, PMRT, Tissue equivalent bolus, Radiodermatitis, Local control

## Abstract

**Purpose:**

To assess the impact and optimize the prescription of tissue-equivalent bolus in postmastectomy radiotherapy (PMRT), we compared the use of different bolus regimens tailored by skin involvement status.

**Methods:**

Patients with breast cancer who required PMRT were recruited (NCT01925651) and classified into two groups: standard-risk (SR, without skin involvement) and high-risk (HR, with skin involvement). SR was randomized between no bolus or 5 mm-bolus on alternate days and HR between 5 mm-bolus on alternate days or daily. Conventional fractionation (50.4 Gy; 1.8 Gy/daily) was used. Acute skin toxicity was evaluated blindly and the radiodermatitis-specific toxicity index [rads-TI] calculated. Subsequently, patients were followed up to assess oncologic outcomes, focusing on chest wall (CW) local control.

**Results:**

Fifty-eight patients were enrolled (34 SR and 24 HR). Baseline characteristics were similar between arms within the same risk group. Overall, maximal radiodermatitis rates were 29.4 % (G2) and 15.7 % (G3). In the SR group, no difference existed in G2 radiodermatitis incidence between the subgroups (p = 0.70) and no G3 events occurred. In the HR group, incidences of G2 (100 % vs 44.5 %, p = 0.01) and G3 radiodermatitis (70 % vs 11.1 %, p = 0.02) were higher with daily bolus. After adjusting for confounders, the daily bolus had a higher incidence of G2 (p = 0.03), G3 radiodermatitis (p = 0.04), and worse rads-TI (p < 0.01). After a median follow-up of 6.2 years, the 5-year local control was 95.8 % (95 %CI: 88.2 %–100 %) in the SR and 91.7 % (95 %CI: 77.3 %–100 %) in the HR groups. Per risk group, there was no difference in local control between the SR (p = 0.90) or the HR bolus regimens (p = 0.70).

**Conclusion:**

Daily 5 mm bolus prescription significantly increased the overall toxicity burden. In this preliminary study, within the same risk group, no detriment in CW local control was detected with less intense bolus regimens (SR: no bolus; HR: alternate-days bolus). Additionally, the rads-TI was able to distinguish overall radiodermatitis burden.

## Highlights

• Alternate-days bolus may be the most appropriated regimen to increase the absorbed dose in the superficial regions of the CW.
• Due to the excess of G3 radiodermatitis events (70 %), the daily 5 mm-bolus should be avoided in PMRT for NIBC.
• Toxicity-related treatment interruptions are avoided if the bolus is promptly discontinued when a G3 toxicity occurs.
• The proposed *rads-TI* was able to capture the toxicity burden during the entire course of radiotherapy.

## Introduction

Occult residual disease is responsible for locoregional recurrence (chest wall [CW] + regional nodes) after breast cancer treatment. Postoperative radiotherapy (RT) provides a proportional reduction of these failures by a factor of three, which may ultimately reduce breast cancer mortality, especially in node-positive patients [1], [2], [3].

After mastectomy, adequate irradiation of the CW region is technically challenging due to multiple factors, including anatomical alterations from the removal of the affected breast [4] and proximity to adjacent organs (heart, ipsilateral lung, and contralateral breast) [5], [6], [7], [8]. In addition, the superficial location of CW soft tissues coincides with the initial dose buildup region, which is defined as the region between the surface and the point of the maximum dose. Since modern mega-voltage X-rays became available in the late 1950s [9], slower dose buildup has favored the treatment of deep-seated sites (skin-sparing effect) [10]; however, concerns regarding their suitability for treating superficial targets have arisen.

In clinical practice, a tissue equivalent material (called ‘buildup bolus’ or simply ‘bolus’) is frequently placed on top of the skin to increase the dose to the superficial tissues of the CW. This classical technical solution has been utilized for decades, but there is a paucity of evidence to guide the optimal trade-off between the increased dose absorbed in superficial tissues and toxicity. In this regard, recent retrospective studies [11], [12], [13], [14], [15] failed to demonstrate a local control benefit of bolus compared with no bolus. However, since bolus prescription in daily practice is intrinsically influenced by physicians’ perception of high-risk factors, such as skin involvement, lymphovascular space invasion (LVSI), and positive margins, these studies are limited by important imbalances between the intervention (bolus) and control (no bolus) subgroups [11], [12], [13], [14]. In addition, these researchers evaluated the impact of the intervention (bolus) without consistently defining which bolus regimen [12], [14], [15] was used (i.e., thickness, frequency) and if or when the bolus was discontinued earlier owing to skin toxicity.

In this context, we designed a randomized controlled trial to evaluate the impact of the use of a CW bolus in two distinct settings of post-mastectomy radiotherapy (PMRT): standard risk of recurrence (negative skin) and high risk of recurrence (positive skin). In the first group, no bolus was compared to the use of bolus on alternate days. In the high-risk group, bolus on alternate days was compared with daily bolus. The main outcome was acute skin toxicity, and the secondary outcomes were treatment interruptions and oncologic outcomes, focusing on the CW component of locoregional control.

## Methods

### Study design

The inclusion criteria were histological diagnosis of breast cancer, mastectomy (with or without immediate breast reconstruction), and indication of postoperative RT. The exclusion criteria were a Karnofsky Performance Scale (KPS) score <70 % and previous ipsilateral thoracic/cervical irradiation. To increase the generalizability of the results to the male population [16], the protocol was revised to allow inclusion of male patients that meet all other criteria. The institutional review board (Comitê de Ética em Pesquisa em Seres Humanos da Fundação Antônio Prudente) approved the study design and the use of patient information without name or facial identification. All patients signed informed consent forms before enrollment in the study. This study was registered at the Clinicaltrial.gov, identifier NCT01925651.

### Treatment protocol

All patients with positive lymph nodes (clinical or pathological), tumor greater than 5.0 cm (T3), or tumor invading the CW and/or skin (T4) received CW irradiation via opposed tangent photon fields (6 MV). Those with four or more positive axillary nodes received an additional supraclavicular photon field. The bolus device (5 mm thickness) was placed covering the whole chest wall target (from the anterior median line to the mid-axillary line).

All patients received 50.4 Gy (28 × 1.8 Gy, once a day) to the CW and, when indicated, 45 Gy (25 × 1.8 Gy, once a day) to the nodal drainage using a two or three-field technique [17]. No additional scar boost or electron field for the coverage of internal mammary nodes were used. The use of planning computed tomography (CT) was left to the discretion of the attending physician and was decided before randomization. Chemotherapy was delivered following the institutional protocol (combination of doxorubicin and cyclophosphamide followed by paclitaxel with addition of trastuzumab for HER2-positive patients). After RT, patients with hormone receptor-positive disease received antiestrogen medication. Radiotherapy was initiated at least 10 days after the last adjuvant chemotherapy cycle or surgery (if received neoadjuvant chemotherapy was received).

### Risk stratification and randomization

The patients were classified into two risk groups: a) standard risk (no skin involvement) and b) high risk (skin involvement). Skin involvement was defined as clinical (cT4b) and/or pathological (pT4b or ypT4b; with extension to the dermal lymphatics) disease. None of the patients had inflammatory breast cancer (IBC, T4d).

The standard-risk (SR) group patients were randomized between no bolus or 5 mm bolus used on alternate days. To avoid patients with positive skin randomized being treated without bolus, the high-risk (HR) group was randomized between two more intense bolus regimens (5 mm alternate days vs 5 mm daily/continuous days). Randomization of each risk group was performed by balancing the patient’s body mass index (BMI; the weight in kilograms divided by the square of the height in meters) < 30 kg/m^2^ and ≥ 30 kg/m^2^ (obesity) [18], [19], [20].

### Endpoints

The primary study endpoint was the occurrence of acute radiodermatitis in the CW area, which was graded using the RTOG/EORTC toxicity scale [21] (Supplementary Material S1). Bolus use (alternate or continuous) was discontinued if the patient developed G3 radiodermatitis. Weekly toxicity evaluations were performed by two nurses with experience in the diagnosis and management of radiodermatitis who were blinded to the assigned bolus regimen (single-blinded design). Patients who developed G3+ radiodermatitis (during or after the end of treatment) were evaluated in two additional occasions after the end of RT (at the first and third post-treatment weeks).

The granular information of the weekly toxicity grades was used to compute the radiodermatitis-specific toxicity index (rads-TI), adapted from the work of Rogatko et al. [22]. The rads-TI accounts for all observed weekly radiodermatitis grades during the whole RT course and is intended to refine the toxicity evaluation when compared to the simple rate of maximum toxicity or the averaged toxicity. The integer portion represents the maximum toxicity, and the fractional portion represents the additional weekly toxicity experienced over the duration of treatment. Multiple toxicities of the same intensity (grade) yield a rads-TI score that is slightly less than that generated by a single event of the next higher grade. Importantly, when several patients are compared with respect to toxicity, the rads-TI preserves their ranking [23]. The rads-TI statistic is presented in the Supplementary Material S2.

The secondary endpoints were the rate of definitive treatment interruption and survival outcomes (local control, metastasis-free interval, and overall survival). Local control was defined as failure in the CW area, excluding regional failure. Metastatic cases detected after trial registration (before or during RT delivery) were only evaluated in the toxicity analyses.

### Statistical analysis

The power (sample size) calculation is presented in Supplementary Material S3. The characteristics of the subgroups within each randomization arm were compared using the Fisher’s exact test.

Time to event endpoints (incidences of G1, G2, and G3 radiodermatitis, oncologic outcomes) were calculated from the date of RT start (T_0_), and the rates were estimated using the Kaplan-Meier method. The relative risks (RR) for G2 and G3 radiodermatitis were calculated using a quasi-Poisson regression model adjusted for possible confounding factors (age, BMI, diabetes mellitus, smoking history, and RT technique). Furthermore, we summarized the repeated measurements in a single measure, such as rads-TI, and the comparison between subgroups was performed using the probabilistic index model (PIM) [24], [25], [26], [27], [28], [29], in which the probability index (PI), a measure of relative effect, is modeled as a function of covariates. The PI represents the probability that the outcome (e.g., radiodermatitis) of a patient randomly sampled from a specific bolus subgroup (alternate days or daily) is greater than the outcome of another patient randomly sampled from the no-bolus subgroup, conditional on the covariate values of both patients, and a PI equal to 0.5 indicates that both subgroups have similar toxicity score distribution. The PI with Wald-type 95 % confidence interval (95 %CI) was reported with p-values based on the Wald statistic.

Toxicity analyses were performed using the ‘*pim’* package [30] and survival analyses were performed using the *‘survival’* and ‘*survminer’* packages, implemented in R software version 4.1.0 (R Foundation for Statistical Computing, Vienna, Austria) [31].

## Results

### Patients

**Fig. 1:**
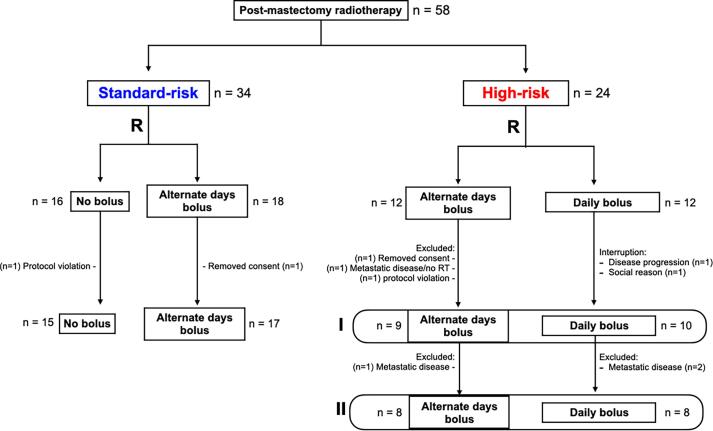
CONSORT diagram. Risk stratification before randomizations: standard risk [SR] group = no skin involvement (n = 32); high-risk [HR] group = positive skin involvement (n = 19). All SR patients evaluated for toxicity and oncologic outcomes (n = 32). HR I: evaluated for toxicity (n = 19). HR II: evaluated for oncologic outcomes (n = 16).

During the recruitment period (August 2013 to November 2013), 58 patients were enrolled. Thirty-four patients without skin involvement (SR) and 24 patients with skin involvement (HR) proceeded to the respective bolus randomization arm (Fig. 1). Two patients withdrew consent before RT, two patients had a protocol violation (received other bolus regimens), and three had treatment interruptions. In total, 51 and 48 patients were included in the toxicity and oncologic outcome analyses, respectively.

The median age of the patients was 48 years (interquartile range: 43.7–61.2), and 35.3 % had a BMI > 30 kg/m^2^. Breast reconstruction (immediate prosthetic implant) was performed in 21.6 % of the patients (none in the HR subgroups). Thirty-one percent had a positive smoking history, and 13.7 % had diabetes mellitus as a comorbidity; all baseline characteristics were similar between each subgroup within the same risk group and are described in the Table 1.

**Table 1:** Patients, disease and treatment characteristics (overall and per risk-group).

Characteristic	Overall	Standard-risk			High-risk		
		No bolus	Alternate days bolus	*p*-value*	Alternate days bolus	Daily bolus	*p*-value*
**Age**							
≤ 50 years old	26 (51%)	8 (53.3%)	8 (47.1%)	>0.99	4 (44.4%)	6 (60%)	0.66
> 50 years old	25 (49%)	7 (46.7%)	9 (52.9%)		5 (55.6%)	4 (40%)	
**BMI**							
≤ 30 kg/m^2^	33 (64.7%)	10 (66.7%)	11 (64.7%)	>0.99	5 (55.6%)	7 (70%)	0.65
> 30 kg/m^2^	18 (35.3%)	5 (33.3%)	6 (35.3%)		4 (44.4%)	3 (30%)	
**Sex**							
Female	50 (98%)	15 (100%)	17 (100%)	>0.99	9 (100%)	9 (90%)	>0.99
Male	1 (2%)	0 (0%)	0 (0%)		0 (0%)	1 (10%)	
**Race**							
White	28 (54.9%)	9 (69.2%)	6 (35.3%)	0.14	7 (77.8%)	6 (60%)	0.63
Non-white	21 (41.1%)	4 (30.8%)	11 (64.7%)		2 (22.2%)	4 (40%)	
**Breast reconstruction**							
No	40 (78.4%)	9 (60%)	12 (70.6%)	0.71	9 (100%)	10 (100%)	>0.99
Yes	11 (21.6%)	6 (40%)	5 (29.4%)		0 (0%)	0 (0%)	
**Breast Side**							
Right	27 (52.9%)	9 (60%)	9 (52.9%)	0.73	4 (44.4%)	5 (50%)	>0.99
Left	24 (47.1%)	6 (40%)	8 (47.1%)		5 (55.6%)	5 (50%)	
**DM**							
No	44 (86.3%)	15 (100%)	14 (82.4%)	0.23	8 (88.9%)	7 (70%)	0.59
Yes	7 (13.7%)	0 (0%)	3 (17.6%)		1 (11.1%)	3 (30%)	
**Smoking history**							
Never	35 (68.6%)	11 (73.3%)	10 (58.8%)	0.47	7 (77.8%)	7 (70%)	>0.99
Ever	16 (31.4%)	4 (26.7%)	7 (41.2%)		2 (22.2%)	3 (30%)	
**RT technique**							
Conventional	21 (41.2%)	7 (46.7%)	5 (29.4%)	0.47	4 (44.5%)	5 (50%)	>0.99
CT-planning	30 (58.8%)	8 (53.3%)	12 (70.6%)		5 (55.5%)	5 (50%)	
**RT target**							
CW	15 (29.4%)	7 (46.7%)	6 (35.3%)	0.72	1 (11.1%)	1 (10%)	>0.99
CW + nodes	36 (70.6%)	8 (53.3%)	11 (64.7%)		8 (88.9)	9 (90%)	
**RT timing**							
AM	16 (31.4%)	3 (20%)	3 (17.6%)	>0.99	6 (66.7%)	4 (40%)	0.37
PM	35 (68.6%)	12 (80%)	14 (82.4%)		3 (33.3%)	6 (60%)	
**Skin involvement**							
No	32 (62.7%)	15 (100%)	17 (100%)	>0.99	0 (0%)	0 (0%)	>0.99
Yes	19 (37.3%)	0 (0%)	0 (0%)		9 (100%)	10 (100%)	
**pT stage**							
T0–T2	32 (62.7%)	10 (66.7%)	11 (64.7%)	>0.99	6 (66.7%)	5 (50%)	0.65
T3–T4	18 (35.3%)	5 (33.3%)	5 (29.4%)		3 (33.3%)	5 (50%)	
NA	1 (2.0%)	0 (0%)	1 (5.9%)		0 (0%)	0 (0%)	
**pN stage**							
pCR/pN0/pN1	35 (68.6%)	11 (73.3%)	11 (64.7%)	0.71	6 (66.7%)	7 (70%)	>0.99
pN2/pN3	16 (31.4%)	4 (26.7%)	6 (35.3%)		3 (33.3%)	3 (30%)	
**pAJCC stage**							
pCR/p II	30 (58.8%)	9 (60.0%)	10 (58.8%)	>0.99	5 (55.6%)	5 (60%)	>0.99
p III-IV	21 (41.2%)	6 (40.0%)	7 (41.2%)		4 (44.4%)	5 (40%)	
**Distant metastasis**							
No	48 (94.1%)	15 (100%)	17 (100%)	>0.99	8 (88.9%)	8 (80%)	>0.99
Yes	3 (5.9%)	0 (0%)	0 (0%)		1 (11.1%)	2 (20%)	
**Margins**							
Negative	51 (100%)	15 (100%)	17 (100%)	>0.99	9 (100%)	10 (100%)	>0.99
Positive	0 (0%)	0 (0%)	0 (0%)		0 (0%)	0 (0%)	
**LVI**							
No	28 (54.9%)	8 (53.3%)	9 (53.0%)	>0.99	6 (66.7%)	5 (50%)	0.65
Yes	23 (45.1%)	7 (46.7%)	8 (47.0%)		3 (33.3%)	5 (50%)	
**Histology**							
IDC	44 (86.3%)	14 (93.3%)	15 (88.2%)	>0.99	9 (100%)	6 (60%)	0.09
Non-IDC	7 (13.7%)	1 (6.7%)	2 (11.8%)		0 (0%)	4 (40%)	
**Grade**							
1–2	24 (47.0%)	9 (60.0%)	9 (53.0%)	0.73	1 (11.1%)	5 (50%)	0.14
3	27 (53.0%)	6 (40.0%)	8 (47.0%)		8 (88.9%)	5 (50%)	
**Subtype**							
Non-triple negative	46 (90.2%)	14 (93.3%)	15 (88.2%)	>0.99	8 (88.9%)	9 (90%)	>0.99
Triple negative	5 (9.8%)	1 (6.7%)	2 (11.8%)		1 (11.1%)	1 (10%)	
**Chemotherapy timing**							
Neoadjuvant	33 (64.7%)	10 (58.8%)	6 (40%)	0.48	9 (100%)	8 (80%)	0.47
Adjuvant	18 (35.3%)	7 (41.2%)	9 (60%)		0 (0%)	2 (20%)	
**Taxane use**							
No	10 (19.6%)	2 (13.3%)	6 (35.3%)	0.23	0 (0%)	2 (20%)	0.48
Yes	41 (80.4%)	13 (86.7%)	11 (64.7%)		9 (100%)	8 (80%)	

BMI: body-mass index. DM: diabetes mellitus. RT: radiotherapy. CT: computed tomography. CW: chest wall. AJCC: American Joint Committee on Cancer. IDC: invasive ductal carcinoma. LVI: lymphovascular invasion. NA: not available.

* Fisher’s Exact test.

### Toxicity

**Fig. 2:**
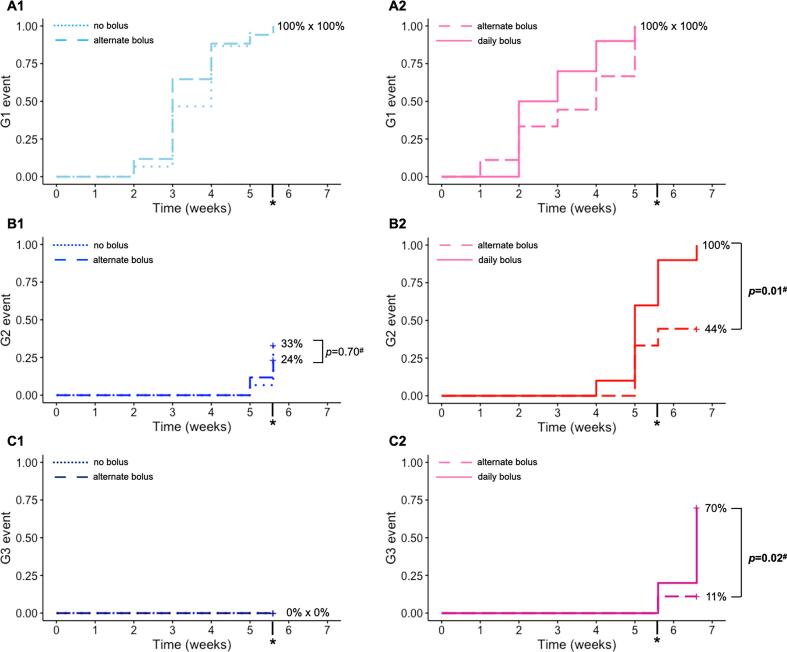
Occurrence of acute toxicity events per study subgroup: (A1) SR grade 1 (light blue); (B1) SR grade 2 (blue); (C1) SR grade 3 (dark blue); (A2) HR grade 1 (pink); (A2) HR grade 2 (red); (A3) HR grade 3. Dashed line: no bolus. Interrupted line: alternate days bolus. Continuous line (daily bolus). *end of treatment (last RT fraction). ^#^Fisher's exact test.

All patients developed at least G1 radiodermatitis (Fig. 2A1 and 2A2), and the maximal acute toxicity observed in the overall population was 54.9 % G1, 29.4 % G2, and 15.7 % G3. No G4 events occurred. Five G3 toxicity events (62.5 %) occurred after the last fraction of RT. Three patients had the bolus discontinued after achieving grade 3 toxicity during the last week of treatment, all in the high-risk subgroups (alternate-day bolus: one patient; daily bolus: two patients).

In the standard-risk group, there was no difference in the incidence of G2 radiodermatitis at the end of RT between the no-bolus and the alternate-day bolus subgroups (33.3 % vs 23.5 %, p = 0.70, Fig. 2B1). No patient developed G3 event in this group (Fig. 2C1). In the high-risk group, the incidences of G2 radiodermatitis (100 % vs 44.5 %, p = 0.01) and G3 radiodermatitis (70 % vs 11.1 %, p = 0.02) were higher in the daily bolus subgroup than in the alternate-days bolus subgroup (Fig. 2B2 and 2C2).

Comparing the bolus prescriptions, the use of daily (continuous) bolus remained significantly associated with the development of G2 radiodermatitis (RR 3.1; 95 % CI: 1.2–8.2), G3 radiodermatitis (RR 25.8; 95 % CI: 1.3–520.3) and higher rads-TI scores (PI 88.4 %; 95 % CI: 76.3–94.8), after adjusting for confounding variables. There was no statistical difference in any toxicity measures between alternate days bolus and no bolus subgroups (Table 2, Fig. 3, and Supplementary material S4).

**Table 2:** Univariable and multivariable analyses for radiodermatitis.

Endpoint	Bolus regimen	Events/total	RR (unadjusted)	95%CI	p-value	RR (adjusted#)	95%CI	p-value
G2 *	No	6/15	1			1		
	Alternate	15/26	0.92	(0.39-2.20)	0.86	0.93	(0.36-2.38)	0.88
	**Daily**	**10/10**	**2.70**	**(1.15-6.31)**	**0.03**	**3.11**	**(1.18-8.20)**	**0.03**

G3**	No or alternate	1/41	1			1		
	**Daily**	**7/10**	**28.70**	**(4.03-204.33)**	**<0.01**	**25.81**	**(1.28-520.28)**	**0.04**

RR: relative risk. CI: confidence interval. Reference subgroup: no bolus.

* At the end of treatment (5.6 weeks).

** At the end of acute toxicity follow-up visits (8.6 weeks).

# Adjusted by age, BMI, DM, smoking history, and RT technique.

**Fig. 3:**
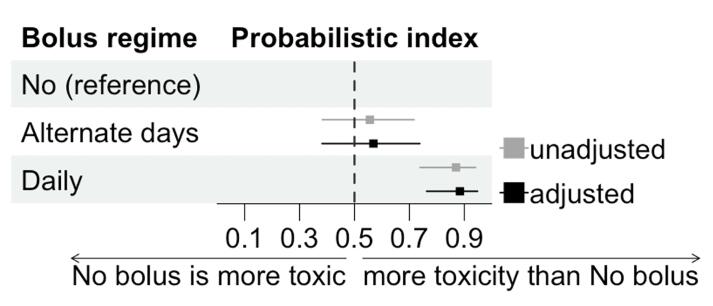
Forest plot representing the probabilistic index (PI) of higher radiodermatitis-specific toxicity index (rads-TI) for each bolus prescription compared to the reference subgroup (no bolus).

### Treatment interruptions

Three patients in the high-risk group (3/24) interrupted treatment definitively: one in the alternate-days subgroup (owing to metastatic progression before RT start) and two in the daily bolus subgroup (one owing to metastatic progression in fraction 5 and another owing to social reasons in fraction 10). As these definitive interruptions occurred early and were not related to toxicity, these three patients were not included in the toxicity analyses.

### Oncologic outcomes

After a median follow-up of 6.2 years, four patients developed local failure in the CW region (one patient in each of the four arms). The overall 5-year local control rate was 95.8 % (95 % CI: 88.2 %–100 %) in standard-risk patients and 91.7 % (95 % CI: 77.3 %–100 %) in high-risk patients. Local recurrences were the first site of failure in two patients and occurred after metastatic progression in the other two (one and three months after the date of metastatic progression).

**Fig. 4:**
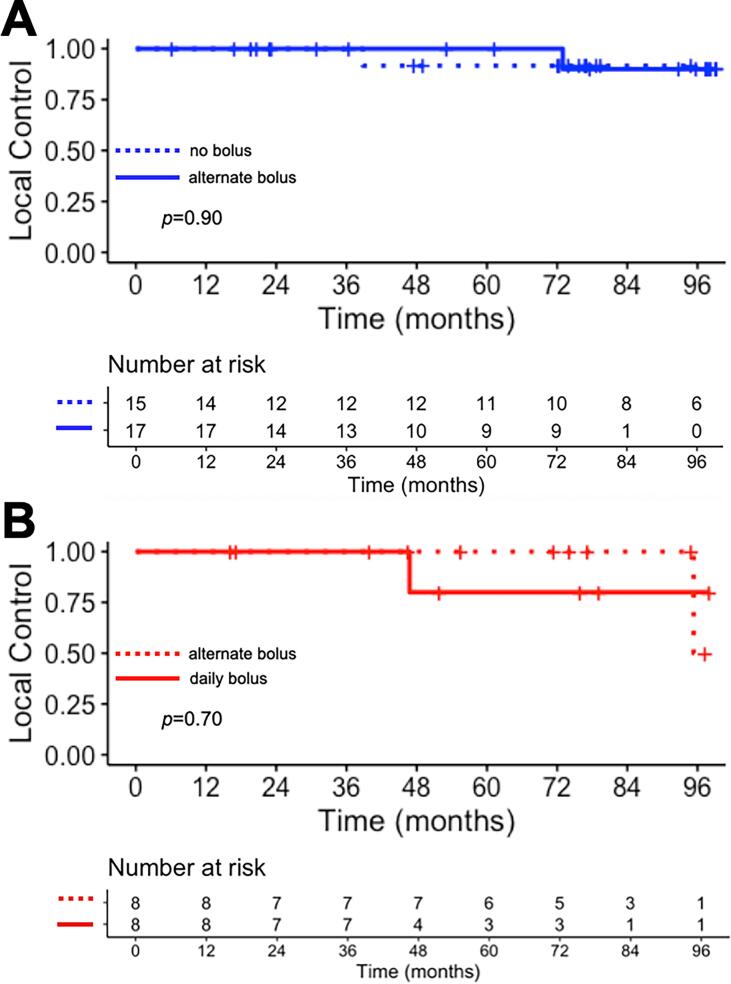
Local control (chest wall) estimated using Kaplan-Meier method. (A) SR group randomization. (B) HR group randomization. Log-rank p-value provided.

Per randomization arm, there was no difference in CW local control between the standard-risk subgroups (no bolus versus alternate days bolus, p = 0.90, Fig. 4A) and between the high-risk subgroups (alternate days bolus versus daily bolus, p = 0.70; Fig. 4B). The locations of local failures are presented in Supplementary material S5. No differences in metastasis-free interval or overall survival were found between the SR-no bolus and SR-alternate bolus subgroups or between the HR-alternate bolus and HR-daily bolus subgroups (Supplementary material S6).

## Discussion

In this first randomized controlled trial investigating the impact of tissue equivalent bolus in PMRT, we applied a risk-based approach for bolus prescription to better understand the overall toxicity burden on the patient by adding this device. For SR (negative skin) patients, both bolus intensities (none or alternate days) showed similar toxicity profiles. However, for HR (positive skin) patients, daily bolus caused a sixfold increase in G3 toxicity incidence compared with a less intense alternate day regimen. These findings, in conjunction with the absence of detectable differences in CW local control between each prescription, might set a potential limit for the use of more intense bolus regimens outside clinical trials.

In the present study, the daily bolus prescription clearly exceeded the tolerance of normal CW skin to irradiation. Patients treated with this intensified regimen (daily bolus subgroup; 6 MV photons) had a 70 % rate of G3 radiodermatitis (moist desquamation). Notably, most of these events (>60 %) occurred a few days after the end of treatment, explaining the lower rates previously observed in retrospective cohorts [12], [13], [15]. In the only prospective evaluation focusing on skin toxicity (not randomized), Pignol et al. [32] found 41 % moist desquamation with daily bolus in an observational cohort treated with 6MV photons (52 % of cases) or higher energy (48 % of cases). Counterintuitively, they found that 6 MV was associated with less radiodermatitis than higher energy photons (Odds Ratio: 0.46, p = 0.04). However, the authors acknowledged an important imbalance: bolus (5 mm or 10 mm) was used in the higher energy subgroup, but most of the patients treated with the lower energy (6 MV) did not use bolus. Importantly, in our study, we demonstrated that prompt interruption of the bolus at the time of the G3 event avoids toxicity-related RT interruptions, which were hypothesized to potentially reduce CW local control [11], [12], [15].

Additionally, we applied an adapted version of the toxicity index (TI) introduced by Rogatko et al. [22], the rads-TI. As an example, this index allows the differentiation of two patients with maximum grade 3 but distinctive experiences: one that developed radiodermatitis early in the RT course (weekly grades: 0/1/2/2/3) and another that experienced radiodermatitis only towards the end of treatment (weekly grades: 0/0/0/1/3). In our cohort, the probability of worse rads-TI was significantly higher with daily bolus (p < 0.01) than with no bolus. After this demonstration of an increased level of skin toxicity detected by multiple measurements, it is valid to review the rationale and available clinical data supporting bolus use.

Historically, the notion that the addition of a buildup bolus is necessary was based on central axis depth dose measurements [33], although these are known to under-estimate the superficial dose for oblique-incidence beams, as indicated by a series of reports [34], [35], [36]. Clinical support started with small retrospective reports on inflammatory breast cancer (diffuse skin involvement) treatment [37], [38], [39] that suggested an association between slower dose buildup and decreased CW local control. In 1976, Barker et al. [37] described, for non-operative cases, a 34 % (14/41) local failure rate when treating with more superficial 250 keV X-rays, compared with 48 % failure (10/21) when using ^60^Co (even with bolus). In a subsequent study from the same institution directly confronting skin reactions and local control, Thoms Jr et al. in 1989 [38] showed 13 % (3/23) CW failure after PMRT in patients who developed brisk erythema (G2) or moist desquamation (G3), compared with 30 % (7/23) of patients with less than brisk erythema (^60^Co or electron beam).

For non-inflammatory breast cancer, seven retrospective analyses on the impact of the addition of bolus on CW local control were published between 1993 and 2021 [11], [12], [13], [14], [15], [39], [40]. Three of these studies suggested a lower failure rate (crude) with bolus: Hong Kong [41] (22.2 % vs 0 %, not significant [NS]), Boston [40] (5 % vs 4 %), and Maidstone [13] (1.87 % vs 0.99 %, NS). In contrast, four studies showed a higher failure rate in the bolus subgroups: Ontario [12] (13.46 % vs 3.43 %, statistically significant [SS]), Pennsylvania [15] (7.54 % vs 5.66 %, NS), British Columbia-Canada [14] (1.91 % vs 0.94 %, NS), and Sydney [11] (9.09 % vs 8.69 %, NS. The wide range of effect sizes – ranging from 22 % absolute benefit to 10 % absolute detriment in local control with the use of bolus - highlights the complexity of this subject [41], [42].

In essence, these previous studies, compared a high-risk group with bolus (multiple regimens mixed) versus a lower risk group without bolus. Instead of proving the absence of benefit of bolus, they simply indicate that the CW local control of high-risk patients ‘with bolus’ is similar to that of lower-risk patients (without bolus). Although some hypotheses can be generated, they certainly do not provide information regarding the optimal bolus prescription for each risk group to be used in clinical practice, which remains ultimately guided by physician preference [43], [44]. In this scenario, the CW local control rates (secondary outcome) presented in our study are the first controlled data that objectively indicate that a difference in CW local control might not be clinically meaningful with the less intense bolus regimens for each risk group (SR: no bolus; HR: alternate days bolus). Further refinement of the risk stratification and additional accumulation of controlled data are imperative to define the role of bolus.

The 6.2-year median follow-up of the present study is expected to cover more than 70 % of local recurrences [45]; however, the power to detect small differences between subgroups was limited by the modest sample size and rareness of CW recurrences after PMRT with the current treatment paradigm. Other limitations of the present study are the absence of in vivo dosimetry data and the sub-utilization of more intense staging exams to exclude metastatic cases more accurately (6 cases were found to be metastatic during treatment, and three of these had the treatment interrupted due to disease progression).

Notwithstanding, our report provides relevant clinical insights: a) due to the excess of G3 radiodermatitis events (70 %), daily 5 mm-bolus (or more intense, such as daily 10 mm) should be avoided in PMRT for non-inflammatory breast cancer outside clinical trial; b) alternate days 5 mm bolus has a toxicity profile comparable to no bolus and may be the most appropriated regimen when increased absorbed dose is desired in the superficial regions of the CW; and c) careful monitoring of G3 radiodermatitis events allowing prompt discontinuation of bolus can avoid toxicity-related treatment interruptions, which could potentially reduce local control. Additionally, we proposed an adapted form of the toxicity index (rads-TI), which accounts for the toxicity burden during the entire course of RT and preserves the ranking of patients, allowing more refined comparisons between subgroups. Of note, the present study was conducted in a setting where all patients were treated with conventional 1.8 Gy daily fractions and 40 % without CT planning. Although our findings are not applicable to other types of energies (electrons and protons), it is plausible to assume that concerns regarding the coverage of superficial tissues with mega-voltage photons remain independently of the use of CT planning or of the more recent retracted fractionations [46], [47].

Despite the very high overall CW local control rate observed, the CW recurrence incidence in the HR group was twice that in the SR group (8.3 % vs 4.2 % in five years), indicating that patients with positive skin might be considered the preferred population for subsequent controlled trials testing the benefit of bolus and/or other methods of dose escalation. Finally, our results should not be applied to ‘very high’ risk patients, which include inflammatory breast cancer (IBC) or positive margins, where controlled studies are also lacking.

In summary, based on a risk-tailored approach, we provided initial controlled evidence to support the use of less intensive bolus regimens and reduce the toxicity burden to patients affected by breast cancer who underwent mastectomy and require radiotherapy. In this scenario, the preferred prescriptions may consist of: (A) no bolus for patients without skin involvement; and (B) alternate-days 5 mm bolus for patients with positive skin involvement. Daily 5 mm (or more intense) bolus should be avoided in PMRT for non-inflammatory breast cancer (NIBC).

## Funding

None.

## Data sharing statement

Research data are stored in an institutional repository and will be shared upon request to the corresponding author.

## Declaration of Competing Interest

The authors declare that they have no known competing financial interests or personal relationships that could have appeared to influence the work reported in this paper.

## References

[1] Whelan T.J., Julian J., Wright J., Jadad A.R., Levine M.L. (2000). Does loco regional radiation therapy improve survival in breast cancer? A meta-analysis. J Clin Oncol.

[2] Clarke M., Collins R., Darby S. (2005). Effects of radiotherapy and of differences in the extent of surgery for early breast cancer on local recurrence and 15-year survival: an overview of the randomised trials. Lancet.

[3] McGale P., Taylor C., Correa C. (2014). Effect of radiotherapy after mastectomy and axillary surgery on 10-year recurrence and 20-year breast cancer mortality: meta-analysis of individual patient data for 8135 women in 22 randomised trials. Lancet.

[4] Kaidar-Person O., Offersen B.V., Boersma L. (2021). Tricks and tips for target volume definition and delineation in breast cancer: lessons learned from ESTRO breast courses. Radiother Oncol.

[5] Darby S.C., Ewertz M., McGale P. (2013). Risk of ischemic heart disease in women after radiotherapy for breast cancer. N Engl J Med.

[6] Erven K., Weltens C., Nackaerts K. (2012). Changes in pulmonary function up to 10 years after locoregional breast irradiation. Int J Radiat Oncol Biol Phys.

[7] Prochazka M., Hall P., Gagliardi G. (2005). Ionizing radiation and tobacco use increases the risk of a subsequent lung carcinoma in women with breast cancer: case-only design. J Clin Oncol.

[8] Boice J.D., Harvey E.B., Blettner M., Stovall M., Flannery J.T. (1992). Cancer in the contralateral breast after radiotherapy for breast cancer. N Engl J Med.

[9] Ginzton E.L., Mallory K.B., Kaplan H.S. (1957). The Stanford medical linear accelerator. I. Design and development. Stanford Med Bull.

[10] Velkley D.E., Manson D.J., Purdy J.A., Oliver G.D. (1975). Build-up region of mega-voltage photon radiation sources. Med Phys.

[11] Tieu M.T., Graham P., Browne L., Chin Y.S. (2011). The effect of adjuvant postmastectomy radiotherapy bolus technique on local recurrence. Int J Radiat Oncol Biol Phys.

[12] Yap M.L., Tieu M., Sappiatzer J. (2018). Outcomes in patients treated with post-mastectomy chest wall radiotherapy without the routine use of bolus. Clin Oncol (R Coll Radiol).

[13] Turner J.Y., Zeniou A., Williams A., Jyothirmayi. (2016). Technique and outcome of post-mastectomy adjuvant chest wall radiotherapy – the role of tissue-equivalent bolus in reducing risk of local recurrence. Br J Radiol.

[14] Nichol A., Narinesingh D., Raman S. (2021). The effect of bolus on local control for patients treated with mastectomy and radiation therapy. Int J Radiat Oncol Biol Phys.

[15] Abel S., Renz P., Trombetta M. (2017). Local failure and acute radiodermatological toxicity in patients undergoing radiation therapy with and without postmastectomy chest wall bolus: is bolus ever necessary?. Pract Radiat Oncol.

[16] Corrigan KL, Mainwaring W, Miller AB, et al. Exclusion of man from randomized phase III breast cancer clinical trials. Oncologist 2020;25(6):e990-992. (PMID: 32272505). 10.1634/theoncologist.2019-0871 PMC7288651 32272505

[17] Zeidan Y.H., Habib J.G., Ameye L. (2018). Postmastectomy radiation therapy in women with T1–T2 tumors and 1 to 3 positive lympho nodes: analysis of the breast international group 02–98 trial. Int J Radiat Oncol Biol Phys.

[18] Fossaluza V., Diniz J.B., Pereira B.B., Miguel E.C., Pereira C.A.B. (2009). Sequential allocation to balance prognostic factors in a psychiatric clinical trial. Clinics (Sao Paulo).

[19] World Health Organization (1995). Physical status: the use and interpretation of anthropometry. Report of a WHO Expert Committee. World Health Organ Tech Rep Ser.

[20] Xie Y., Wang Q., Hu T. (2021). Risk factors related to acute radiation dermatitis in breast cancer patients after radiotherapy: a systematic review and meta-analysis. Front Oncol.

[21] Cox J.D., Stetz J., Pajak T.F. (1995). Toxicity criteria of the Radiation Therapy Oncology Group (RTOG) and the European Organization for Research and Treatment of Cancer (EORTC). Int J Radiat Oncol Biol Phys.

[22] Rogatko A., Babb J.S., Wang H., Slifker M.J., Hudes G.R. (2004). Patient characteristics compete with dose as predictors of acute treatment toxicity in early phase clinical trials. Clin Cancer Res.

[23] Razaee Z.S., Amini A.A., Diniz M.A. (2021). On the properties of the toxicity index and its statistical efficiency. Stat Med.

[24] Gresham G., Diniz M.A., Razaee Z.S. (2020). Evaluating treatment tolerability in cancer clinical trials using the toxicity index. J Natl Cancer Inst.

[25] Fay M.P., Malinovsky Y. (2018). Confidence intervals of the Mann-Whitney parameter that are compatible with the Wilcoxon-Mann-Whitney test. Stat Med.

[26] De Neve J., Thas O. (2017). A Mann-Whitney type effect measure of interaction for factorial designs. Commun Stat-Theory Methods.

[27] De Neve J., Thas O. (2015). A regression framework for rank tests based on the probabilistinc index model. J Am Stat Assoc.

[28] De Neve J., Thas O., Ottoy J.P. (2013). Goodness-of-fit methods for probabilistic index models. Commun Stat-Theory Methods.

[29] Thas O., De Neve J., Clement L., Ottoy J.P. (2012). Probabilistic index models. J R Stat Soc Ser B (Stat Methodol).

[30] Meys J, De Neve J, Sabbe N, Amorim GGA. pim: Fit Probabilistic Index Models. 2020; R package version 2.0.2. https://CRAN.R-project.org/package=pim.

[31] R Core Team. R: A language and environment for statistical computing. Vienna, Austria: R Foundation for Statistical Computing; 2020. Available at: https://www.R-project.org/; last accessed on 07/10/2022.

[32] Pignol J.P., Vu T.T., Mitera G. (2015). Prospective evaluation of severe skin toxicity and pain during postmastectomy radiation therapy. Int J Radiat Oncol Biol Phys.

[33] Saunders J.E., Price R.H., Horsley R.J. (1968). Central axis depth doses for a constant source-tumour distance. Br J Radiol.

[34] Hughes H.A. (1959). Measurements of superficial absorbed dose with 2 MV x rays used at glancing angles. Br J Radiol.

[35] Bush R.S., Johns H.E. (1962). The measurement of build-up on curved surfaces exposed to Co60 and Cs137 beams. Am J Roentgenol Radium Ther Nucl Med.

[36] Jackson W. (1971). Surface effects of high-energy X rays at oblique incidence. Br J Radiol.

[37] Barker J.L., Nelson A.J., Montague E.D. (1976). Inflammatory carcinoma of the breast. Radiology.

[38] Thoms W.W., McNeese M.D., Fletcher G.H. (1989). Multimodal treatment for inflammatory breast cancer. Int J Radiat Oncol Biol Phys.

[39] Soong I.S., Yau T.K., Ho C.M. (2004). Post-mastectomy radiotherapy after immediate autologous breast reconstruction in primary treatment of breast cancers. Clin Oncol (R Coll Radiol).

[40] Uematsu M., Bornstein B.A., Recht A. (1993). Long-term results of post-operative radiation therapy following mastectomy with or without chemotherapy in stage I-III breast cancer. Int J Radiat Oncol Biol Phys.

[41] Kaidar-Person O., Dahn H.M., Nichol A.M. (2021). A Delphi study and international consensus recommendations: the use of bolus in the setting of postmastectomy radiation therapy for early breast cancer. Radiother Oncol.

[42] Dahn H.M., Boersma L.J., de Ruysscher D. (2021). The use of bolus in postmastetomy radiation therapy for breast cancer: a systematic review. Crit Rev Oncol Hematol.

[43] Vu T.T.T., Pignol J.P., Rakovitch E., Spayne J., Paszat L. (2007). Variability in radiation oncologists’ opinion on the indication of a bolus in post-mastectomy radiotherapy: an international survey. Clin Oncol (R Coll Radiol).

[44] Thomas K., Rahimi A., Spangler A., Anderson J., Garwood D. (2014). Radiation practice patterns among United States radiation oncologists for postmastectomy breast reconstruction and oncoplastic breast reduction. Pract Radiat Oncol.

[45] Taghian A., Jeong J.H., Mamounas E. (2004). Patterns of locoregional failure in patients with operable breast cancer treated by mastectomy and adjuvant chemotherapy with or without tamoxifen and without radiotherapy: results from five National Surgical Adjuvant Breast and Bowel Project randomized clinical trials. J Clin Oncol.

[46] Haviland J.S., Owen J.R., Dewar J.A. (2013). The UK standardisation of breast radiotherapy (START) trials of radiotherapy hypofractionation for treatment of early breast cancer: 10-year follow-up results of two randomised controlled trials. Lancet Oncol.

[47] Brunt A.M., Haviland J.S., Wheatley D. (2020). Hypofractionated breast radiotherapy for 1 week versus 3 weeks (FAST-Forward): 5-year efficacy and late normal tissue effects results from a multicentre, non-inferiority, randomised, phase 3 trial. Lancet.

