# Supplementary material for: Randomized clinical trial of tissue equivalent bolus prescription in postmastectomy radiotherapy stratified by skin involvement status
Source: Clin Transl Radiat Oncol. 2022 Dec 21;39:100570. doi: 10.1016/j.ctro.2022.100570 (PMC9803916; doi:10.1016/j.ctro.2022.100570)
Supplement: Supplementary data 1 [file mmc1.docx]

Supplementary Material for:

**Randomized clinical trial of tissue equivalent bolus prescription in postmastectomy radiotherapy tailored by skin involvement status**

**Table of Contents**

| **Supplementary material S1.** Toxicity evaluation. | 2 |
| --- | --- |
| **Supplementary material S1.** The rads-TI statistic. | 3 |
| **Supplementary material S2.** Power considerations. | 4 |
| **Supplementary material S3.** Violin plot of radiodermatitis-specific toxicity index (rads-TI) for each bolus prescription. | 5 |
| **Supplementary material S4.** Location of the CW failures. Blue ‘X’ (failures in the standard-risk group), Red ‘X’ (failures in the high-risk group). | 6 |
| **Supplementary material S5.** Additional oncologic outcomes. A1: metastasis free interval (standard-risk group). B1: overall survival (standard-risk group). A2: metastasis free interval (high-risk group). B2: overall survival (high-risk group). *Log-rank test. | 7 |
|  |  |
| **Author contributions** | 8 |

**Supplementary material S1.** **Toxicity evaluation.**

The severity levels were as follows:

grade 0 (G0) no change over baseline;

grade one (G1) faint erythema/dry desquamation;

grade two (G2) tender/bright erythema, patchy moist desquamation;

grade three (G3) confluent moist desquamation; and

grade four (G4) ulceration, hemorrhage, or necrosis.

Reference:

Cox JD, Stetz J, Pajak TF. Toxicity criteria of the Radiation Therapy Oncology Group (RTOG) and the European Organization for Research and Treatment of Cancer (EORTC). Int J Radiat Oncol Biol Phys 1995;31(5):1341-1346. (PMID: 7713792)

**Supplementary material S2.** **The rads-TI statistic.**

The adapted rads-TI index is a weighted sum of the ordered radiodermatitis grades observed over time. Let be $X_{1}\geq X_{2}\geq\ldots{\geq X}_{j}$ the sequence of toxicity grades in a subject’s toxicity weekly evaluations of radiodermatitis in descending order. The statistic is calculated as follows:

$$rads-TI=\sum_{i=1}^{J} \frac{X_{i}}{\prod_{l<i} (1+X_{l})},$$

where $J$ represents the number of measurements observed over time. The resulting toxicity index statistics quantifies the toxicities experienced by individual patient over the trial period.

Reference:

Rogatko A, Babb JS, Wang H, Slifker MJ, Hudes GR. Patient characteristics compete with dose as predictors of acute treatment toxicity in early phase clinical trials. Clin Cancer Res 2004;10(14):5645-51. (PMID: 15269136)

**Supplementary material S3.** **Power considerations.**

The main hypothesis to be tested is whether there is a difference in grade 2 radiodermatitis at the end of treatment between no bolus and bolus treatment. The sample size was calculated based on incidence of grade 2 radiodermatitis of 40% in no bolus arm and 80% in the bolus arm*, that is, an effect size of 40%. The required sample size for each group using a one-sided Fisher’s exact test with 80% power at 5% significance level was 23 patients (46 patients in both groups). This was inflated to 29 patients (**58 patients** in both groups) to account for missing data.

* Limited controlled data available after extensive literature review.

**Supplementary material S4. Violin plot of radiodermatitis-specific toxicity index (rads-TI) for each bolus prescription.**

**
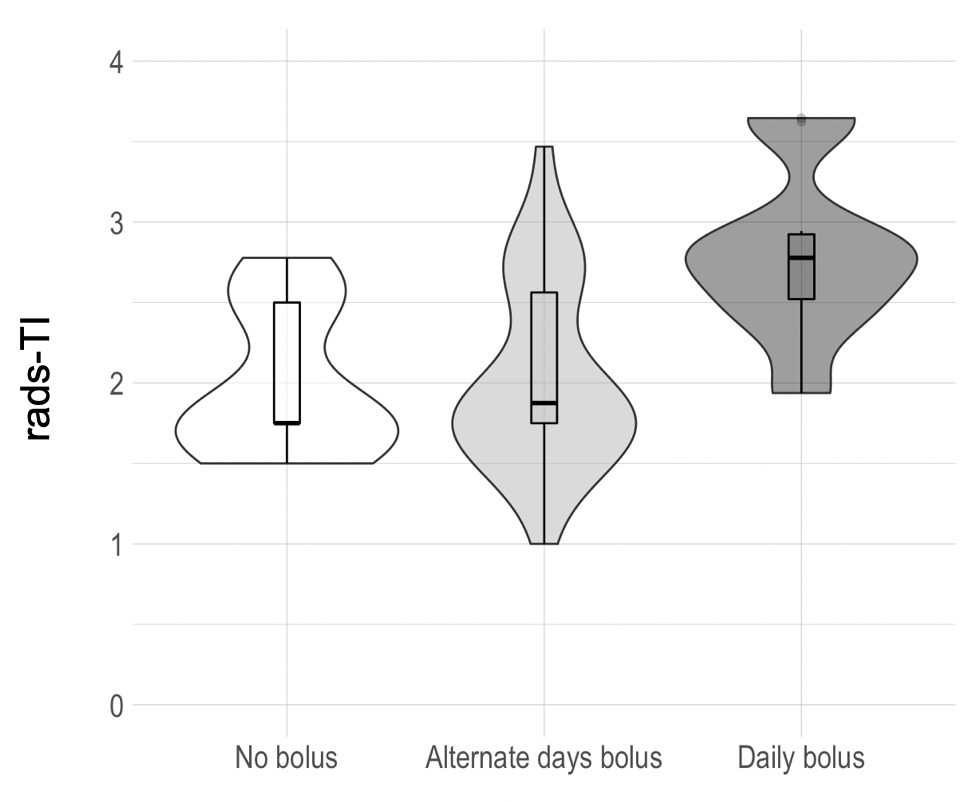
**

**Supplementary material S5. Location of the CW failures. Blue ‘X’ (failures in the standard-risk group), Red ‘X’ (failures in the high-risk group).**

**
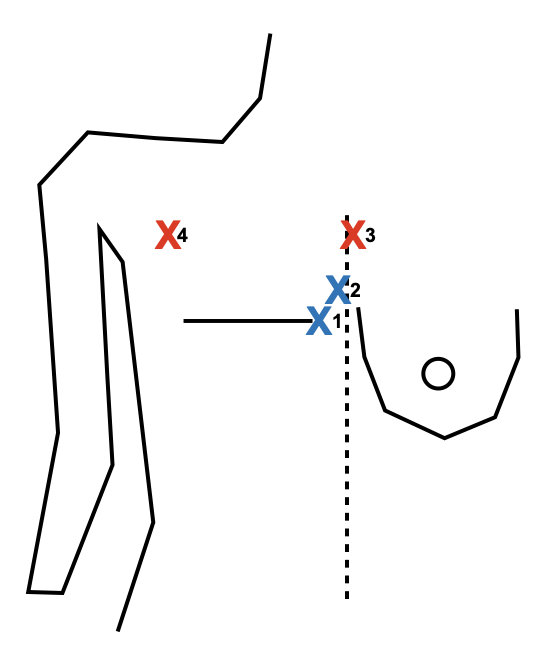
**

**Supplementary material S6. Additional oncologic outcomes. A1: metastasis free interval (standard-risk group). B1: overall survival (standard-risk group). A2: metastasis free interval (high-risk group). B2: overall survival (high-risk group). *Log-rank test.**

**
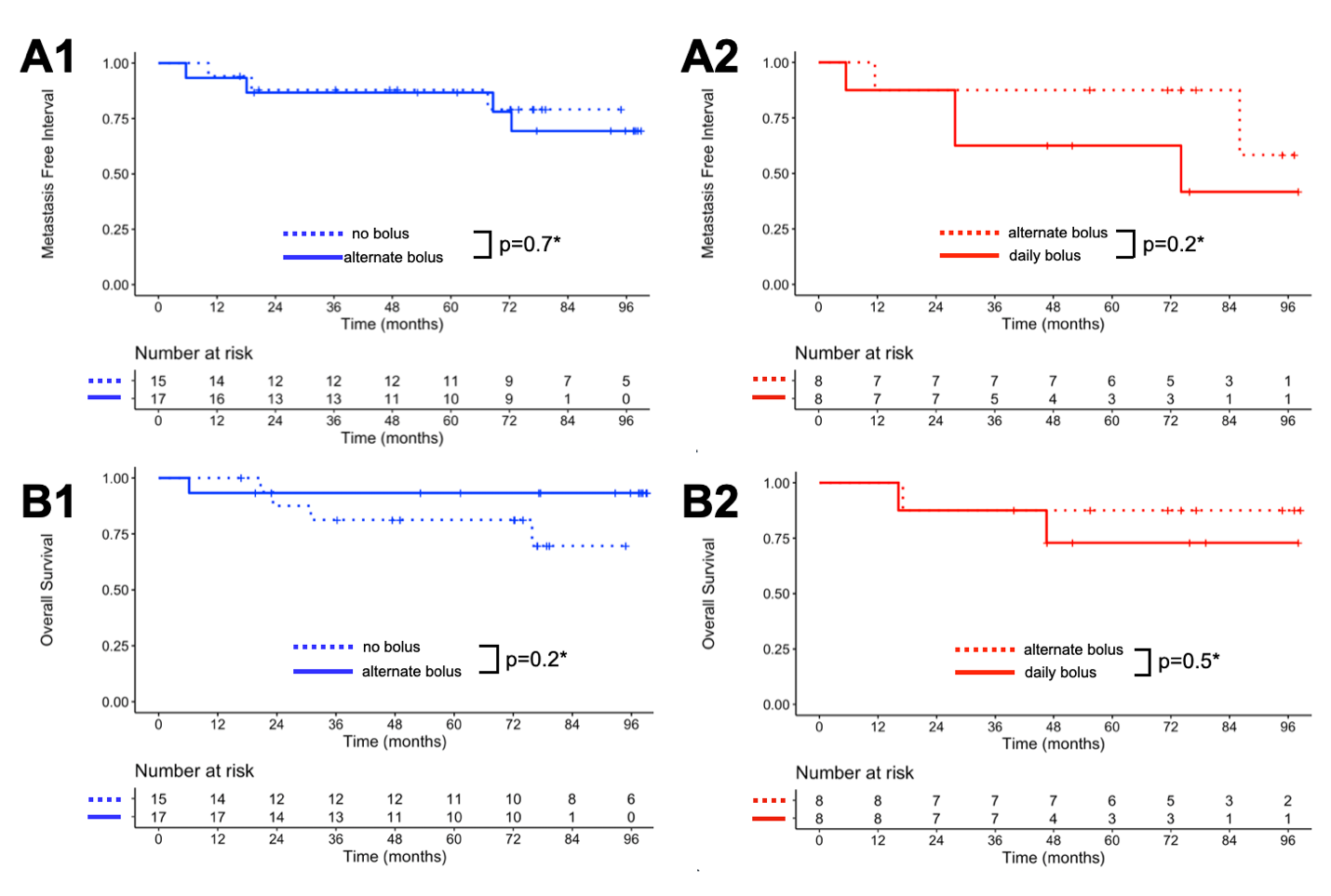
**

**Author Contributions**

LGS, MACM, and MJLG designed the study. LGS, MJLG, AM, GB and VFC supervised data abstraction and vouch for the data. LGS and VFC analyzed the data and vouch for it. LGS, MJLG and VFC wrote the first draft of the manuscript. All authors approved the final version of the manuscript and decided to publish the paper.
